# Medico-Political Union's Action

**Published:** 1919-01-11

**Authors:** 


### A WHOLE-TIME SALARIED MEDICAL SERVICE.

### MEDICO-POLITICAL UNION'S ACTION.

The Medico-Political Union, having considered the scheme for a whole-time salaried service submitted to them as receiving attention in certain Parliamentary quarters, passed the following resolution : "The Council of the Medico-Political Union declares its unalterable opposition to any whole-time salaried service for the medical profession. Further, that any such service would be detrimental to the welfare of the community as well as the interests of the profession."

The Panel Medico-Political Union was registered under the Trade Union Acts in 1915, and in June last the word "Panel" was eliminated. Every duly qualified member of the profession, whether consultant or general practitioner, is eligible for membership, and the general secretary is Dr. A. Welply, 14 Gray's Inn Square, London, W.C. The annual subscription to the Union is one guinea, for which a copy of the official journal, post free, is sent each week. Being a trade union, the Union is unable to use any portion of its ordinary funds for political objects. The Union is in fact one of medical men registered under the Trade Union Acts 1871 and 1876.

The following has been issued to the profession by the Union :

### The Scheme for a Whole-time Medical Service.

There has come to the knowledge of the Council of the Medico-Political Union the full outline of a proposed scheme for a whole-time medical service, and after reading it and realising that it is being carefully canvassed amongst those general practitioners who are now in the Army and observing further the huge problems lying far outside any scope of inquiry by the R.A.M.C., one can but imagine that the Government sits in the prompter's box taking vast interest in how the "show" goes. There is other circumstantial evidence which satisfies the Council that the information is reliable.

The scheme is to be a whole-time medical service, and practitioners entering it are to be graded into five classes. Although this is to be a civil service, military rank is now so universally understood that we may conveniently detail these classes together with the five military ranks to which they correspond. Class 5, lieutenant; Class 4, captain; Class 3, major; Class 2, lieutenant-colonel; and Class 1, equal to a colonel's rank.

The commencing pay for the 5th class equal to the lieutenant is £400 a year, with promotion to the 4th class at £500, 3rd class at £750, 2nd class at £1,000, and 1st class equal to the full colonel at £1,500 a year.

The 2nd and 1st class will remain at these amounts, but for the lower classes there will be yearly increments, for the 5th class of £15 a year until a maximum of £500 is reached, for the 4th class a rise of £25 a year until the 3rd or major's class, when the annual increase will be £50 a year, till £1,000 is reached. Unless examinations are passed the 5th rank man will never get above his £500 a year.

There must be four years' interval between the 5th and 4th classes and eight years' interval between both the 4th and 3rd, and 3rd and 2nd, from which they will be chosen to the highest class at £1,500 a year. There will be no necessity for a vacancy to exist before a man can qualify for a higher class and pay, at any rate up to the 3rd or major class.

Promotion will be by examination for each class.

The higher classes will deal chiefly with administrative work and with the giving of consultative advice.

There will be no expenses to be paid out of the medical man's salary, everything, drugs, appliances, clinics, and travelling expenses, will be paid for by the Government. Men will be expected to look after between two and three thousand patients or persons' healths. It will not be necessary to live in the big houses at present in vogue, any sized house and in any adjacent locality will do as long as it has telephonic communication. On the argument that either a patient is fit for work or unfit, so he will be expected to attend a morning surgery, evening surgery will be discontinued, and less visits paid than is the case at present.

Work out of hours—that is to say after five o'clock—will be taken by the juniors, who will take duty in rotation.

Localities will be mapped out with exact boundaries, and in these areas all residents, whether rich or poor, will be entitled to medical benefit.

All hospitals will be taken over by the Government.

There will probably be no compensation for the capital value of practices except in cases of unusual hardship; but those entering the scheme will have pensions at sixty, though perhaps they may go on practising after that date, such pensions to be two-thirds of their salary.

As a commencement men who have been in practice for many years and are otherwise knowledgeable will be enabled to enter the service at a higher class than the lowest or Class 5.

Full consultant and specialist services will be provided; men will be appointed to them after taking a special course and passing an examination in that particular work. They will receive higher pay. Study leave up to six months will be granted. Holiday leave will be the same as in the Civil Services, a month per year.

All local lay control will be done away with, the service being under a central board of commissioners which will consist of—

(1) Members appointed by the Government from outside the medical profession. (2) Members appointed by the Government from men at the head of the medical profession. (3) Members elected by those medical men serving in the scheme.

It is thought by this method to make the best use of the medical profession. What the general public will say about it is another matter, and for a time there will be ample scope for those medical men who do not wish to come into the scheme, but in the future young men leaving the hospitals would in some numbers pass into this civil service. The scheme has been mainly considered as offering a solution to the demobilisation of the thousands of medical men now in the Army, to many of whom it may at first sight appear very tempting.

#### Some Remarks on the Scheme.

It is often said that those who have left their practices will return to find them vanished and worthless, but much evidence was brought forward to show that this had not been the case. Those doctors already returned were finding their practices coming back to them more rapidly than they had expected.

Very careful consideration was given to the whole scheme by the Council of the Medico-Political Union, and they decided to again adhere to the principle of "free choice of doctor," and of opposition to a whole-time

(Continued on page 310, opposite.)

### A Whole-Time Salaried Medical Service.

(*Continued from opposite page.*)

medical service; principles which have been accepted so many times at the meetings and conferences of the Union.

The arguments against such a scheme may be divided into those opposing the main principle of a whole-time medical service and, secondly, debatable points and difficulties in the actual working of the scheme as at present before us.

(1) A whole-time medical service has always been opposed because it is feared that the most intimately human of all professions, with its confidences, its entry into every phase of family life, its sympathy with the families' rejoicings and mournings, their births and deaths, its contact alike with the grossest of human passions, and the petty squabbles and weaknesses of the homes it enters, cannot with kindness be turned into a machine. Such confidences, such secrets and confessions will not be forthcoming to the paid official, here to-day and gone perhaps to-morrow, replacing the friend by the judicial and cold machine, shall we not lose that trust in our decision, that compliance with our advice, that is so important a factor in obtaining a cure? Not only must we treat the disease, but we must treat the patient. Nothing is more astonishing than the unquestioning manner in which our verdict is received to-day; after a brief examination of a man, we tell him he must go to the hospital, and we will operate on him at once; he implicitly obeys us. He knows that we are honourable men of good repute, he knows our house and history, we have been his friend and neighbour for many years, we live in the Market Place, our lives are plain and open for all men to see. And this is what the patient banks on, and with his trust in us, the chance of cure is proportionately increased. Even the bottle of medicine has a symbolic value, far above the cost of the ingredients. Religion itself would die if the sacrament were to be obtained from a penny-in-the-slot machine. (2) "Free choice of doctor" was supported for the above reasons. Educate your doctor, give him all facilities for the latest and most up-to-date aids to his diagnosis and treatment, but to do away with that huge incentive to work, fair competition with his fellow-practitioners, is a most dangerous principle, and liable always to allow the slowest mover to set the pace. (3) Whether there be five or more classes is immaterial. But we should like to know more details as to the method of classifying men, especially the older practitioners, when the scheme is starting. Is age, length of time in practice, past income or examination knowledge to be the main factor? Will the man joining at 55 be a full pensioner at 60? (4) And the pay. Though excellent and attractive to the newly qualified and freed from all professional expenses, is hardly munificent around £700 after 12 years' service, at an age when so many of us indulge in that most expensive hobby of sending our children to decent schools. (5) It is true much money can be saved by moving from a large house into a smaller one, but what of the years of lease yet to run? Again many men have bought or built their own houses, and fitted them up accordingly, and look to the selling of such together with their practices to realise in cash a life-time's work and investment. Under such a scheme, men would have to go where the service wanted them, and after settling to a smaller house, a fresh move and another might be necessary, with the extraordinary inconsequence any army or naval officer's wife can tell of. (6) And at a time when the profession is told to take a larger hand in public affairs, local, municipal, in the County Councils and even Imperial Parliament, such a retrograde movement to a smaller house, to sink to a nonentity, one of a public service, to become perhaps a shifting population, will hardly tend to a realisation of medical men's aspirations. It may be cheaper to do without one's own car, but the joy-ride will go too. (7) The absence of evening surgery would be a vast boon to medical men, but is it possible? In the morning the working man works, and the woman attends to the children and home, and neither will want to leave these duties instead of seeing the doctor in the evening at the usual time. (8) The difficulty of defining areas will be great and more difficult still in correctly apportioning an equal share of work to each man. Take London to start with, and compare this difficulty in the East End and the West. In the one a tremendously congested population, in the other a sparse population, but requiring much fidgety attendance; or, on the other hand, will they refuse in bulk to attend a clinic? What then? (9) All hospitals to be taken over by the Government, a procedure impossible to criticise without more detail, but one may ask, would such a scheme dismiss those at present holding appointments there? Nevertheless, changes in hospital arrangements are urgently called for. It is ridiculous that the profession's voluntary services, originally a matter of charity to the necessitous poor, should be exploited to complete Government services, whether it be the insured person, the school child, or the discharged disabled soldier. (10) If any scheme is brought forward with the hope of enlisting the support of general practitioners as a whole, it must have definite and sufficient compensation for the capital value of the practices men have worked for and invested their moneys in. Nor should a definite scale of annuities and death payments be difficult to work out on an insurance basis. (11) Men are so deeply distrustful of the term "Commissioners" after their dealings with those of the National Health Insurance that they will be very shy of entering into new contracts in the future, unless they can see all the cards on the table first.

The attitude of omnipotence, of legal verbosity, of prejudged hostility to deputations, would suggest that the profession must rely on more weighty arguments than words to help them in further schemes and settlements with the Government.

Many good points are obvious in this whole-time scheme, but they can equally well be obtained by other methods.

The Council of the Medico-Political Union recognise that changes in medical service are inevitable; they go further and would do all in their power to welcome a more efficient service, giving greater scope for assisting the lower classes and on wider grounds: they note a great opportunity for more preventive and educational medicine to lessen the horribly large proportion of inefficients observable when the nation as a whole strips for its medical examination at the recruiting centres.

We wish to see the national physique which has been dwarfed by our slums, poisoned by overcrowding, stunted by unnatural work and unwholesome feeding and ignorance, restored to the fine type of our broad-shouldered cousins who visit us to-day from the open air of the dominions. We look to see the medical man sitting supreme in all councils where the nation's health is under debate, unhampered by political intrigues, by the huge voting power of commercial interests and the Approved Societies. Let us first examine our position by looking at a quite reasonable parallel.

Suppose for a moment that nationalisation of the railways is to be carried out and under a vastly changed method as well, *i.e.* the motive power is to be changed to electricity; further an entirely new system of the payment of wages is to be brought in. Now this is to be ruled, and ruled autocratically, beyond the jurisdiction of the common law, by Commissioners in London, one member of whom had been a railway servant some years ago, with local committees, one in ten of whom are railway men, the rest to be representatives of the public using the railways, insurance agents, landowners, County Councillors, educationalists, with a fair sprinkling of cranks of most denominations!

Would any politician in his senses suggest such a scheme to the members of the Railwaymen's Union, to say nothing of the rest of the railway officials?

Yet this is our position to-day, and as ours is a far more technical trade than that of the railway man, so should our trade union position be so much proportionately greater as to be absolutely unassailable.

Changes are about to take place, and a strong Trade Union managed throughout by general practitioners and those who will be most affected by such changes, will be the best argument in support of our wishes, our independence and our whole future outlook as medical men.

A. WELPLY, General Secretary.

